# Data of protein-RNA binding sites

**DOI:** 10.1016/j.dib.2016.12.041

**Published:** 2016-12-29

**Authors:** Wook Lee, Byungkyu Park, Daesik Choi, Kyungsook Han

**Affiliations:** Department of Computer Science and Engineering, Inha University, Incheon, South Korea

**Keywords:** Protein-RNA interactions, Binding sites, Prediction

## Abstract

Despite the increasing number of protein-RNA complexes in structure databases, few data resources have been made available which can be readily used in developing or testing a method for predicting either protein-binding sites in RNA sequences or RNA-binding sites in protein sequences. The problem of predicting protein-binding sites in RNA has received much less attention than the problem of predicting RNA-binding sites in protein. The data presented in this paper are related to the article entitled “PRIdictor: Protein-RNA Interaction predictor” (Tuvshinjargal et al. 2016) [1]. PRIdictor can predict protein-binding sites in RNA as well as RNA-binding sites in protein at the nucleotide- and residue-levels. This paper presents four datasets that were used to test four prediction models of PRIdictor: (1) model RP for predicting protein-binding sites in RNA from protein and RNA sequences, (2) model RaP for predicting protein-binding sites in RNA from RNA sequence alone, (3) model PR for predicting RNA-binding sites in protein from protein and RNA sequences, and (4) model PaR for predicting RNA-binding sites in protein from protein sequence alone. The datasets supplied in this article can be used as a valuable resource to evaluate and compare different methods for predicting protein-RNA binding sites.

**Specifications Table**

**Table:** 

Subject area	Bioinformatics, computational biology
More specific subject area	Molecular structures
Type of data	Text files in XML format
How data was acquired	Protein data bank (PDB) [2] and Nucleic acid-Protein Interaction DataBase (NPIDB) [3]
Data format	Filtered and processed
Experimental factors	
Experimental features	
Data source location	Department of Computer Science and Engineering, Inha University, Incheon, South Korea
Data accessibility	Data is provided with this article.

**Value of the data**

• Few data resources have been available which can be readily used in developing or assessing a method for predicting protein-binding sites in RNA sequences or RNA-binding sites in protein sequences.
• Protein-RNA binding sites at the nucleotide and residue levels can facilitate to develop a new method for predicting protein-RNA binding sites.
• Protein-RNA binding sites provided here can be used as a useful resource to evaluate and compare different methods for predicting protein-binding nucleotides in RNAs and/or RNA-binding residues in proteins.

## 1 Data

The four datasets S1-S4 in XML format can be used to evaluate various methods for predicting: (1) protein-binding nucleotides from protein and RNA sequences, (2) protein-binding nucleotides from RNA sequence alone, (3) RNA-binding amino acids from protein and RNA sequences, and (4) RNA-binding amino acids from protein sequence alone.

## 2 Experimental design, materials and methods

From the Protein Data Bank (PDB) [2], we collected structures of protein-RNA complexes which do not include ribosomal RNAs and were determined by X-ray crystallography with a resolution ≤3.0 Å.

As of September 2013, there were a total of 542 protein-RNA complexes, which contained 546 protein-RNA sequence pairs between 376 protein sequences and 439 RNA sequences.

We defined a protein-RNA binding site using three types of protein-RNA interactions (hydrogen bonds, water bridges and hydrophobic interactions). A nucleotide (or amino acid) involved in at least one of the interactions was classified as a protein-binding (or RNA-binding) site. For each of the protein–RNA complexes from PDB, we obtained the three types of interactions from the Nucleic acid–Protein Interaction DataBase (NPIDB) [3] and incorporated them into the RNA and protein sequences.

In order to reduce overlap between training and test datasets, we ran CD-HIT-EST on the RNA sequences and selected RNA sequences with a similarity of 80% or lower from other RNA sequences and constructed test datasets S1 and S2 for models RP and RaP [1], respectively. The datasets S1 and S2 have same RNA sequences, but have the following differences:

1. Protein sequences were included in the dataset S1 only.
2. In the dataset S2, protein-binding sites in a same RNA sequence with different protein partners were incorporated in the RNA sequence.

The dataset S1 contains 130 protein sequences and 155 RNA sequences with 1848 protein-binding nucleotides and 4631 non-binding nucleotides. The dataset S2 contains 155 RNA sequences with 1795 protein-binding nucleotides and 4235 non-binding nucleotides.

The test datasets S3 and S4 for models PR and PaR were constructed in a similar way. The dataset S3 contains 44 RNA sequences and 46 protein sequences with 923 RNA-binding residues and 7578 non-binding residues. The dataset S4 contains 49 protein sequences with 1349 RNA-binding residues and 11,217 non-binding residues.
