# Supplementary material for: Data of protein-RNA binding sites
Source: Data Brief. 2016 Dec 29;10:561–3. doi: 10.1016/j.dib.2016.12.041 (PMC5219607; doi:10.1016/j.dib.2016.12.041)
Supplement: Supplementary file 1 — Supplementary material [file mmc1.pdf]

Conflict of Interest Disclosure

We have no conflict of interest in relation to this article.
